# Proviral load does not discriminate patients with Human T-Cell Leukemia/Lymphoma (ATLL) from HTLV-1 carriers with a history of Strongyloidiasis

**DOI:** 10.1186/1742-4690-8-S1-A253

**Published:** 2011-06-06

**Authors:** Giovanni López, Elsa González, Daniel Clark, Michael Talledo, Carolina Alvarez, Kristien Verdonck, Angélica Terashima, Eduardo Gotuzzo

**Affiliations:** 1 Instituto de Medicina Tropical “Alexander von Humboldt”, Universidad Peruana Cayetano Heredia, Lima, 31, Perú; 2 Facultad de Medicina Alberto Hurtado, Universidad Peruana Cayetano Heredia, Lima, 31, Perú; 3 Laboratorios de Investigación y Desarrollo (LID), Facultad de Ciencias y Filosofía, Universidad Peruana Cayetano Heredia, Lima, 31, Perú

## Background

Studies evidences that high proviral loads (PVL) are crucial for the leukemogenesis in the development of ATLL. PVL seems to be stable for years in each individual, but varies substantially among subjects. Previous reports suggest that co-infection of HTLV-1 carriers with S. stercoralis may shorten ATLL onset, thus generating a hypothetical “intermediate state”. We explored this hypothesis by comparing the PVL of ATLL patients and HTLV-1/Strongyloides co-infected individuals.

## Methods

We determined PVL in three groups: (1) HTLV-1 asymptomatic carriers without a history of Strongyloidiasis [AC] (n=8); (2) virus carriers with previous episodes of Strongyloidiasis [SS] (n=8); and (3) patients with ATLL [AT] (n=8). History of Strongyloidiasis was defined by the patient’s report and a positive result with a ELISA. Peripheral blood mononuclear cells (PBMC) were isolated to measure PVL by Sybr-Green quantitative PCR. PVL was expressed as HTLV-1 tax copy number/10^4^ PBMC. Non-parametric tests were used for the statistical analysis.

## Results

The PVL in AT was significantly higher than that in AC [7375±4492SD vs.1698±106SD][p=0.01]. PVL was also higher in SS than in AC [4746±1712SD vs.1698±106SD][p=0.002]. Interestingly, no differences were found between AT and SS [7375±4492SD vs.4746± 1712SD][p=0.16].

## Conclusion

Our results support the hypothesis that HTLV-1/S. stercoralis co-infected subjects are at higher risk of developing ATLL as they seem to share with ATLL patients impaired immunological mechanisms to control virus propagation. Further studies are needed to fully characterize the co-infected condition.

